# Relationship Between Sleep and Behavior in Autism Spectrum Disorder: Exploring the Impact of Sleep Variability

**DOI:** 10.3389/fnins.2020.00211

**Published:** 2020-03-24

**Authors:** Abigail Bangerter, Meenakshi Chatterjee, Nikolay V. Manyakov, Seth Ness, David Lewin, Andrew Skalkin, Matthew Boice, Matthew S. Goodwin, Geraldine Dawson, Robert Hendren, Bennett Leventhal, Frederick Shic, Anna Esbensen, Gahan Pandina

**Affiliations:** ^1^Neuroscience Therapeutic Area, Janssen Research & Development, Titusville, NJ, United States; ^2^Computational Biology, Discovery Sciences, Janssen Research & Development, Spring House, PA, United States; ^3^Computational Biology, Discovery Sciences, Janssen Research & Development, Beerse, Belgium; ^4^Statistically Speaking Consulting, LLC, Chicago, IL, United States; ^5^Department of Health Sciences, Bouvé College of Health Sciences, Northeastern University, Boston, MA, United States; ^6^Department of Psychiatry and Behavioral Sciences, Duke Center for Autism and Brain Development, Duke University School of Medicine, Durham, NC, United States; ^7^Department of Psychiatry, School of Medicine, University of California, San Francisco, San Francisco, CA, United States; ^8^Center for Child Health, Behavior and Development, Seattle Children’s Research Institute, Seattle, WA, United States; ^9^Department of Pediatrics, University of Washington, Seattle, WA, United States; ^10^Division of Developmental and Behavioral Pediatrics, Cincinnati Children’s Hospital Medical Center, Cincinnati, OH, United States; ^11^University of Cincinnati, College of Medicine, Cincinnati, OH, United States

**Keywords:** actigraphy, anxiety, ASD, hyperactivity, irritability, sleep, variability

## Abstract

**Objective:**

The relationship between sleep (caregiver-reported and actigraphy-measured) and other caregiver-reported behaviors in children and adults with autism spectrum disorder (ASD) was examined, including the use of machine learning to identify sleep variables important in predicting anxiety in ASD.

**Methods:**

Caregivers of ASD (*n* = 144) and typically developing (TD) (*n* = 41) participants reported on sleep and other behaviors. ASD participants wore an actigraphy device at nighttime during an 8 or 10-week non-interventional study. Mean and variability of actigraphy measures for ASD participants in the week preceding midpoint and endpoint were calculated and compared with caregiver-reported and clinician-reported symptoms using a mixed effects model. An elastic-net model was developed to examine which sleep measures may drive prediction of anxiety.

**Results:**

Prevalence of caregiver-reported sleep difficulties in ASD was approximately 70% and correlated significantly (*p* < 0.05) with sleep efficiency measured by actigraphy. Mean and variability of actigraphy measures like sleep efficiency and number of awakenings were related significantly (*p* < 0.05) to ASD symptom severity, hyperactivity and anxiety. In the elastic net model, caregiver-reported sleep, and variability of sleep efficiency and awakenings were amongst the important predictors of anxiety.

**Conclusion:**

Caregivers report problems with sleep in the majority of children and adults with ASD. Reported problems and actigraphy measures of sleep, particularly variability, are related to parent reported behaviors. Measuring variability in sleep may prove useful in understanding the relationship between sleep problems and behavior in individuals with ASD. These findings may have implications for both intervention and monitoring outcomes in ASD.

## Introduction

Autism spectrum disorder (ASD) is a neurodevelopmental disorder characterized by impairments in social communication and presence of restrictive and repetitive behaviors and interests (American Psychiatric Association [APA], 2013). Many individuals with ASD have co-occurring mental health problems, such as obsessive/compulsive behaviors, aggression, self-injury, mood swings, hyperactivity and concentration issues, anxiety, and sleep disorders (Anagnostou et al., 2015). These difficulties coexist with core diagnostic symptoms and impact overall functioning, quality of life, and treatment outcomes for individuals with ASD.

The prevalence of sleep problems in ASD is reported to be between 50 and 80% (Malow et al., 2006; Richdale and Schreck, 2009; Hollway et al., 2013). This contrasts with a reported prevalence of around 10–30% in typically developing (TD) individuals (Ivanenko and Gururaj, 2009; Ferrie et al., 2011). Sleep difficulties are amongst the first concerns raised by caregivers of young children with ASD (Humphreys et al., 2013; Deliens et al., 2015). Most studies of sleep and ASD have been carried out with children, with fewer studies being reported in adolescents and adults with ASD. Evidence has shown that sleep problems in ASD start in childhood and persist into adulthood (Tani et al., 2003; Matson et al., 2008; Limoges et al., 2013) and it is likely that sleep problems in children with ASD are more persistent than those in TD children (May et al., 2015). A recent meta-analysis of studies in adults with ASD showed increased prevalence of reported sleep problems compared to TD groups in both subjective and objective measures (Morgan et al., 2019).

Caregiver-reported sleep problems are found to relate to core ASD symptoms (Schreck et al., 2004; Mayes and Calhoun, 2009; Hollway et al., 2013; Adams et al., 2014b; Delahaye et al., 2014). The potential relationship between sleep problems and other co-occurring behaviors in ASD suggests that sleep is an important area of focus (Hollway and Aman, 2011). In particular, there is extensive evidence for the relationship between sleep problems in ASD and internalizing behaviors such as anxiety (Mayes and Calhoun, 2009; Goldman et al., 2011; Hollway et al., 2013; May et al., 2015; Mazurek and Sohl, 2016), as well as externalizing behaviors such as hyperactivity and challenging behaviors such as aggression (Mayes and Calhoun, 2009; Adams et al., 2014b; Cohen et al., 2014; May et al., 2015).

The majority of studies to date focus on caregiver-reported sleep problems and other daytime behaviors, such as ASD symptoms, attention, and aggression. However, relying solely on caregiver-report can be problematic. For example, caregivers may over or under report sleep difficulties as a result of differences in expectations or as a reflection of their stress and coping abilities (Richdale and Schreck, 2009). Common sources or shared method variance can also influence relationships between caregiver-reported sleep and other behavior (Podsakoff et al., 2003). There is some evidence for a relationship between caregiver-reported sleep problems and more objective, actigraphy-based measures of sleep (Goldman et al., 2009; Elrod and Hood, 2015; Veatch et al., 2016). Caregivers are found to be most accurate at reporting sleep onset latency (time taken to get to sleep), but relationships between caregiver-report and other measures of sleep are inconsistent (Lambert et al., 2016).

There is less established evidence for the relationship between sleep measures and daytime behaviors when more objective measures such as polysomnography (PSG) or actigraphy are used. A general relationship between ASD symptoms and severity of sleep disturbance, reported by caregivers, has been established in a number of studies (Limoges et al., 2005, 2013; Allik et al., 2006; Malow et al., 2006). However, other studies fail to find a relationship between sleep features and ASD symptomology (Elia et al., 2000; Hare et al., 2006; Miano et al., 2007). A smaller number of studies find associations between actigraphy measures and caregiver-reported behaviors such as affect (Malow et al., 2006; Mayes et al., 2011; Richdale and Baglin, 2015) and externalizing behavior (Anders et al., 2012; Park et al., 2012; May et al., 2015), regardless of whether caregivers report problems with sleep in individuals with ASD.

The majority of actigraphy sleep studies in ASD report on measures relating to average duration of sleep, sleep onset, or sleep quality (Moore et al., 2017). It is likely that additional measures are important in sleep research. For example, frequent changes in sleep timing may misalign the sleep/wake cycle, which can impact cognitive functioning and health (Phillips et al., 2017). Within the TD population, the impact of regular sleep patterns on daytime functioning has been demonstrated (Taub, 1978; Sano et al., 2015; Phillips et al., 2017), and recent research into correlates of daily intra-individual variability (IIV) in sleep/wake patterns report a relationship between IIV and inattention, anxiety, and depression (Becker et al., 2017; Bei et al., 2017).

Little is known about the relationship between IIV of sleep and mood disturbance in individuals with ASD. Sleep studies focusing on mood and behavior in individuals with ASD are also scarce. In one study (Fletcher et al., 2017) demonstrated a relationship between anxiety symptoms with parent-report and actigraphy-derived sleep measures in school-age children with ASD. Sleep variability in preschoolers with ASD (Anders et al., 2012) and more severely affected adolescents and adults with ASD (Cohen et al., 2018) may impact behavior, but this is a new and relatively unexplored area in sleep studies in ASD.

This study examines the relationship between sleep problems and caregiver-reported behaviors in individuals (aged six years to adult) with ASD, assessed using both objective (actigraphy-based) and subjective (caregiver-reported) measures. We hypothesized that both caregiver-reported and actigraphy measures of sleep would significantly correlate and that each of these measures would associate with ASD symptom severity, internalizing and externalizing behaviors (irritability, hyperactivity, and anxiety).

In addition, given the high levels of anxiety symptoms reported in the ASD population (Rodgers et al., 2012; Matson and Williams, 2014; Russell et al., 2016) together with studies suggesting a relationship between anxiety and sleep in TD individuals (Jansson-Fröjmark and Lindblom, 2008; Chen et al., 2017; Hertenstein et al., 2018), the current study explores the use of machine learning to identify relationships between sleep measures and parent reported anxiety. Using an elastic-net model, we repeatedly trained and tested the model through nested cross-validation to identify a set of sleep variables most informative in predicting anxiety behaviors in ASD.

## Materials and Methods

### Ethical Practices

Institutional Review Boards approved the study protocol and its amendments. The study was conducted in accordance with the ethical principles that have their origin in the Declaration of Helsinki, consistent with Good Clinical Practices and applicable regulatory requirements. Participants, their caregivers (for participants <18 years old), or legally authorized representatives provided written informed consent before participating in the study. Participants provided assent. The study is registered at clinicaltrials.gov, NCT02299700.

### Study Population

The study enrolled 144 males and females aged ≥6 years with a confirmed diagnosis of ASD based on clinical judgment, including the Autism Diagnostic Observation Schedule, 2nd edition (ADOS-2) (Lord et al., 2012).

The study also enrolled 41 TD male and females aged ≥6 years with no identifiable mental disorders per the Diagnostic and Statistical Manual of Mental Disorders – Fourth Edition/Fifth Edition (DSM-4/5) (American Psychiatric Association [APA], 2013), reported no significant medical illness, and scored in the non-clinical range on the Social Communication Questionnaire (Rutter et al., 2003) and Mini-international Neuropsychiatric Diagnostic Interview (Sheehan et al., 1998).

### Study Design

This study was part of a multicenter observational study designed to investigate measures of change in ASD conducted from 06 July 2015 to 14 October 2016 at 9 study sites in the United States. Recruitment was through registries held at sites, word of mouth, and advertising by sites (Ness et al., 2019). The study lasted 8 to 10 weeks, with a screening visit wherein participant eligibility was determined and informed consent forms were reviewed and signed. Participants with ASD and their caregivers (usually parents) attended site visits at baseline, midpoint (4 weeks), and endpoint (8/10 weeks) wherein participants took part in a biosensor task battery (Manfredonia et al., 2018; Manyakov et al., 2018) and caregivers completed online and paper rating scales. Participants with ASD were requested to wear the Ambulatory Monitoring, Inc., Motionlogger^®^ Actigraph (AMI) actigraphy watch every night. The AMI Motionlogger, an FDA-approved Medical Device [510(k): K854030] was used in compliance with its intended use, its output was used strictly for exploratory purposes and was not used to diagnose, treat, or prevent any disease or disorder. TD participants completed caregiver-report rating scales during a single visit. No actigraphy measures were collected for TD participants.

### Caregiver-Reported Scales

**FIGURE 1:**
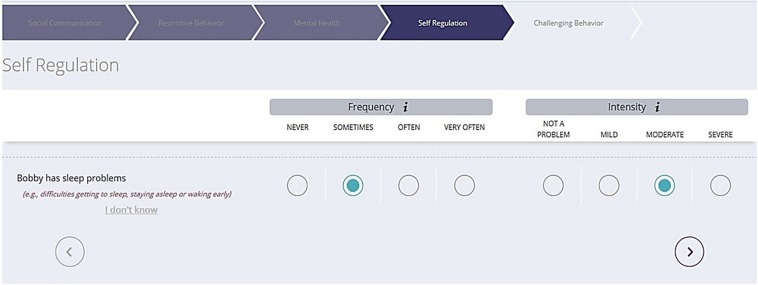
Sample ABI web-based format to capture ASD symptoms. ABI, Autism Behavior Inventory; ASD, autism spectrum disorder.

Caregivers of individuals with ASD completed the following rating scales online and on paper during visits when the task battery was completed:

The Autism Behavior Inventory (ABI) is a new rating scale developed to assess change in core and associated symptoms of ASD (Figure 1; Bangerter et al., 2017). The ABI covers five domains: Core ASD domains of Social Communication; Restricted, Repetitive Behaviors; Social Communication and Associated Domains–Self Regulation; Mental Health; and Challenging Behavior. Of relevance to the present study is the single item pertaining to sleep problems. In addition to the ABI, other established measures described below were used to obtain caregiver-reports on symptom severity and externalizing and internalizing behaviors associated with ASD.

The Social Responsiveness Scale 2 (SRS-2) (Constantino et al., 2003) distinguishes autism spectrum disorder from other psychiatric conditions by identifying the presence and extent of autism symptom severity.

The Child Adolescent Symptom Inventory – Anxiety (CASI-Anx) (Sukhodolsky et al., 2008; Hallett et al., 2013) is a subset of the CASI and uses 21 items related to anxiety. The CASI-Anx was developed for use in TD populations (5–18 years of age), but has been recommended for use in ASD groups (Lecavalier et al., 2014) and used in clinical studies with children and adults (Berry-Kravis et al., 2012).

The Aberrant Behavior Checklist (ABC) (Aman et al., 2004; Aman and Singh, 2017) is a 58-item behavior rating scale used to measure behavior problems among individuals with developmental disabilities across five subscales: Irritability, Lethargy (Social Withdrawal), Stereotypy, Hyperactivity and Non-compliance, and Inappropriate Speech. Items are rated on a four-point Likert scale (ranging from zero [not at all a problem] to three [the problem is severe in degree]), with higher scores indicating more severe problems. Two subscales, Hyperactivity and Irritability, are used as indicators of externalizing behaviors in this study (Aman and Singh, 2017).

### Actigraphy

Participants with ASD were required to wear the AMI actigraphy device – a wristband that measures accelerometry, ambient light, and vibration to determine if the device is attached to the body or not. The AMI was selected due to its data storage capacity, long battery life, and peer-reviewed validation studies demonstrating accuracy (sensitivity and specificity) of the device in sleep-disturbed vs. normal populations (Jean-Louis et al., 2001). Wrist-dependent differences in activity level have no detectable effects on the accuracy of sleep scoring (Sadeh et al., 1994). Participants were requested to wear the AMI on the non-dominant wrist, every night from bedtime until they woke up. Actigraphy data was downloaded at site visits.

### Actigraphy Measures

We performed minute-based sleep activity estimation from actigraphy’s Zero Crossing Mode using the following procedures provided by Action-W (version 2.7.1) software: For each minute, value S = 0.0033^∗^ (1.06 * an4 + 0.54 * an3 + 0.58 * an2 + 0.76 * an1 + 2.3 * a0 + 0.74 * a1 + 0.67 * a2) was estimated, where an4-an1 are activity counts from the prior 4 min, a0 the current minute, and a1 and a2 the following 2 min (Cole et al., 1992) current minute was scored as sleep if S < 1. Results were adjusted based on modifications developed by Jean-Louis et al. in order to further improve sleep detection accuracy (Jean-Louis et al., 2001). Sleep start and end times were identified automatically and all samples were inspected and further manually adjusted as required to account for movement and ambient light information by an expert blind to consequent analysis. Data were discarded from sleep measure estimation and further analyses in instances when it was not possible to clearly identify sleep start and end times.

The following four sleep measures, estimated each night, were selected for comparison with caregiver-report: sleep start, sleep duration, number of awakenings, and sleep efficiency. Sleep start was estimated as time in minutes when a participant went to sleep with respect to midnight. Sleep duration was estimated as time in minutes between sleep start and sleep end. Sleep duration and sleep start were adjusted in line with age group expectations, based on National Sleep Foundation (NSF) guidelines as shown in Table 1 (Hirshkowitz et al., 2015). To adjust sleep duration (or sleep start time) of participants in a given age group, the NSF recommends sleep duration (or recommended sleep start time) be subtracted for that age group from actual sleep duration (or actual sleep start time). The number of awakenings was estimated as number of episodes when scoring changed from sleep to awaking and back to sleep during the night. Sleep efficiency was calculated as the percentage of minutes labeled as sleep within the interval between sleep start and sleep end.

**TABLE 1:** Recommended sleep guidelines by age.

Age group (years)	Recommended sleep	Recommended sleep start (time before midnight)
6–13	600 min (10 h)	−210 min (8:30pm)
14–17	540 min (9 h)	−150 min (9:30pm)
18+	480 min (8 h)	−90 min (10:30pm)

### Data Analyses

Table 2 provides a schematic of the timepoints at which data in this study were collected, including the caregiver-reported scales, actigraphy measures, ADOS and Kauffman Brief Intelligence Test (Kaufman, 2013).

**TABLE 2:** Intervals for assessments of associations of actigraphy sleep measures with caregiver-reported sleep and behaviors.

	Screening	Baseline	Midpoint	Endpoint
Autism Diagnostic Observation Schedule	X			
Kauffman Brief Intelligence Test	X			
Caregiver-reported Scales	X	X	X	X
		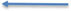	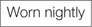	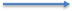
Actigraphy			Midpoint actigraphy week prior to midpoint scales	Endpoint actigraphy week prior to endpoint scales

### Statistical Analyses

Differences in caregiver-reported sleep between ASD and TD groups were assessed using a linear regression model, controlling for sex and age (as intelligence quotient [IQ] was not available for TD group). We calculated the mean and variability of the four different actigraphy-based sleep measures for ASD participants over a one-week time window preceding midpoint and endpoint of the study. Variability of each actigraphy-based sleep measure was calculated in terms of its standard deviation (SD) and coefficient of variation (CV). We then performed association analysis between caregiver-reported sleep or behaviors collected at midpoint and endpoint and actigraphy-based sleep measures using the following mixed effects model:

s⁢c⁢a⁢l⁢e∼(1∣p⁢a⁢r⁢t⁢i⁢c⁢i⁢p⁢a⁢n⁢t)+a⁢c⁢t⁢i⁢g⁢r⁢a⁢p⁢h⁢y+a⁢g⁢e+s⁢e⁢x+I⁢Q

Here *scale* represents a caregiver-reported sleep or behavior, and *actigraphy* represents mean or variability of an actigraphy-based sleep measure. The dependent variables *age*, *sex*, *IQ*, and *actigraphy* comprise the fixed effects in the model. The random effect term is modeled by repeat measures of participants data collected from midpoint and endpoint of the study. Participants’ data was normalized, centered and scaled before performing the above mixed effects model analysis.

A time window of one week was chosen for the purpose of assessing relationships as close as possible to specific visit time points, while at the same time achieving a modest sample size for analysis. To negate the possibility of noise introduced due to technical or inherent random variability over a relatively long interval, mean and variability of actigraphy sleep measures were calculated for only those individuals with ASD who wore their AMI watches for at least 2 nights in the week preceding study midpoint and endpoint. Although our hypotheses were pre-specified and association analyses were confirmatory, we controlled for multiple comparisons in our analyses. A false discovery rate (FDR) of 0.05 was used and the Benjamini & Hochberg procedure was implemented to control for FDR (Benjamini and Hochberg, 1995).

Associations between caregiver-reported sleep and other caregiver-reported scales were analyzed using Spearman correlation incorporating sex, gender, and IQ as covariates for all individuals at baseline, midpoint, or endpoint. To control for multiple comparison, an FDR of 0.05 was applied using the Benjamini & Hochberg procedure.

### Prediction Analyses

Seventeen different features comprised the overall set of predictors (*feature vector x*), and 12 of these features were obtained from actigraphy sleep measures (mean, SD, and CV each for sleep start, sleep duration, number of awakenings, and sleep efficiency). Additional features (which were not based on actigraphy sleep measures) included caregiver-reported sleep obtained from ABI Self-Regulation Sleep and the following four features: age, gender, IQ, and ADOS CSS total scores. The CASI-Anx was used as the dependent variable (response variable *y*). The model was developed using all participants combined from their corresponding midpoint (*n* = 58) and endpoint (*n* = 38) visits for a combined total of 96 participants.

Before training the model, the following pre-processing steps were performed. First, we identified features which had near zero variance. Each feature was flagged as near-zero variance if the percentage of unique values was less than 10% of the total number of samples, and the ratio of the samples having most frequent to second most frequent value was greater than 19 (Kuhn, 2008). No feature had near-zero variance and hence no feature was removed in this step. Second, multicollinearity between different features was tested to prevent unstable model solutions. To do so, we computed a correlation matrix of all 17 predictors and used an iterative method to remove subsets of correlated predictors such that absolute values of all pairwise correlations were below a threshold of 0.7 (Kuhn, 2008). Finally, after removing correlated features, a variance inflation factor (VIF) for each feature was calculated by regressing it against all others to further ensure that all features obtained from the second step had a VIF below a threshold of 10 (Dormann et al., 2013).

We then developed an elastic net model on the data after centering the response variable and standardizing predictors. Elastic net is a penalized linear least square regression analysis technique often used in machine learning as a regularization and variable selection method (Zou and Hastie, 2005). For an α strictly between 0 and 1, and a non-negative parameter λ, elastic net solves the following minimization problem:

$$\min_{\beta_{0},\beta }\left\{\frac{1}{2\,N}\,\sum_{i=1}^{N}{\left(y_{i}-\beta_{0}-x_{i}^{T}\,\beta \right)}^{2}+\lambda \,P_{\alpha }\,\left(\beta \right)\right\},$$

where, Pα(β)=1-α2||β||+22α||β||1=∑j=1p(1-α2βj2+α|βj|)

*y*_*i*_: response variable at observation *i*.

*x*_*i*_: vector of features at observation *i*.

*p*: number of features in *x*_*i*_

*N*: number of observations.

β_0_,β: a scalar and vector of regression coefficients of size *p*.

The function *P*_α_(β) is the elastic net penalty and is a convex combination of least absolute shrinkage and selection operator (lasso) and ridge regression. As α approaches 0, elastic net approaches ridge regression. When α equals 1, elastic net reduces to lasso.

## Results

A total of 144 participants with ASD, the majority of whom were male (77.8%), consistent with the higher male:female ratio in ASD, were enrolled (Loomes et al., 2017). Mean (SD) age of participants was 14.6 (7.83) years. Mean (SD) ADOS total score of participants was 7.6 (1.7), IQ was 99.2 (19.6), and all were verbal. Of the 41 TD participants in the overall study, 34 had completed the ABI sleep item, which was used for comparison in this study. Mean (SD) age of the TD group was 11.4 (5.38) years, and 74% were male.

Table 3 provides demographic details for ASD participants included in correlation analyses at a given time point. Caregivers of 139 individuals with ASD completed scales at baseline. Mean (SD) age of participants at baseline was 14.5 (7.9) years and IQ composite score was 99.0 (19.55). Fifty-eight (42%) participants wore the device with sufficient quality data for two or more nights in the week preceding the midpoint visit. In the week preceding the endpoint visit, 38 (27%) participants wore the device with sufficient quality data for two or more nights. Table 4 shows the distribution of nights for which participants wore the device in the week before midpoint and endpoint.

**TABLE 3:** Demographics of ASD participants by group.

Characteristic	All ASD participants at baseline	Participants wearing AMI watch (for ≥2 nights) over 7 days before midpoint	Participants wearing AMI watch (for ≥2 nights) over 7 days before endpoint
Number of participants	139	58	38
Age (years), mean (SD)	14.5 (7.91)	13 (7.17)	14 (6.01)
Median (range)	12 (6–54)	12 (6–51)	12 (6–32)
Male, n (%)	108 (77.7)	50 (86.2)	30 (78.9)
KBIT-2 IQ composite score, mean (SD)	99.0 (19.55)	98.83 (18.94)	99.16 (18.22)
ADOS CSS Total Score, mean (SD)	7.59 (1.74)	7.24 (1.74)	7.5 (1.81)
Caregiver-reported sleep problems, n (%)	59 (42.45)	23(39.66)	13 (34.21)
ABI sleep, mean (SD)	4.83 (1.19)	4.48 (1.08)	4.54 (1.33)
Sleep duration (min), mean (SD)		480.98 (72.45)	450.1 (81.09)
Sleep start (min), mean (SD)		−81.21 (76.18)	−73.15 (78.27)
Awakenings, mean (SD)		17.5 (6.2)	18.3 (6.69)
Sleep efficiency, mean (SD)		90.99 (4.73)	89.02 (8.12)

*ADOS CSS, Autism Diagnostic Observation Schedule calibrated severity score; AMI, Ambulatory Monitoring, Inc., Motionlogger Actigraph; KBIT-2, Kaufmann Brief Intelligence Test-2 IQ; SD, standard deviation; N, number of ASD participants for each group that was used in correlation analyses.*

**TABLE 4:** Distribution of nights for which participants wore the device.

Number of nights	Participants wearing AMI watch 7 days before midpoint	Participants wearing AMI watch 7 days before endpoint
2	21	14
3	12	5
4	10	6
5	9	9
6	2	4
7	4	0

### Caregiver-Reported Sleep Problems: ASD and TD Group Comparisons

**FIGURE 2:**
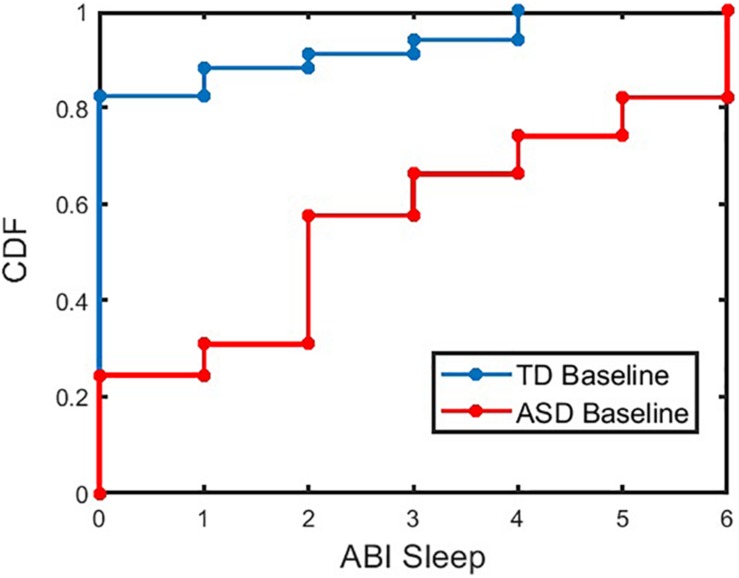
Cumulative Distribution Frequency (CDF) of ABI SR sleep for TD and ASD participants at baseline. The CDF plot for ASD (or TD) indicates the proportion of ASD (or TD) participants with less than or equal to a given value of ABI sleep item. For example, approximately 57% of ASD (or 91% of TD) participants have ABI Sleep score less than or equal to a value of 3. ABI, Autism Behavior Inventory; ASD, autism spectrum disorder; TD, typically developing.

First, caregiver-reported sleep problem differences between ASD and TD participants were investigated before focusing analyses specifically in the ASD sample. Caregiver-reported sleep measured by the ABI sleep item between these two groups was significantly different (*p* < 0.001 for TD and ASD at baseline). Figure 2 shows the cumulative distribution of the ABI sleep item for TD and ASD participants. The percentages of caregivers reporting more than mild current sleep problems (≥ 3 on the ABI sleep item) for TD and ASD participants at baseline were 11.8% and 69.1%, respectively (*p* < 0.001, chi-squared test). Further, when split into age groups, both older individuals (≥13 years of age) and younger individuals (<13 years of age) showed significant difference (*p* < 0.001) in the ABI sleep item between TD and ASD categories for both age groups at baseline. The percentage of caregivers reporting more than mild sleep problems for TD and ASD participants in the older age group at baseline were 9.1% and 64.7%, respectively (*p* < 0.001, chi-squared test). In the younger age group, the percentage of caregivers reporting more than mild sleep problems for TD and ASD participants at baseline was 13.0% and 73.2%, respectively (*p* < 0.001, chi-squared test). In addition, we compared the ABI caregiver reported sleep problems between ASD adults (age ≥ 18 years) and children (age < 18 years) using a linear regression model controlled for age, gender, and IQ. No significant difference was observed between the two groups at baseline (*p* = 0.15), midpoint (*p* = 0.5), or endpoint (*p* = 0.2). The mean (SD) of caregiver reported sleep problems in ASD adults and children were 2.69 (2.15) and 2.63 (2.17), respectively.

### Relationship Between Caregiver-Reported Sleep and Actigraphy: ASD

Participants with ASD exhibited variations in actigraphy measures across nightly measurements. Table 5 shows the relationship between caregiver-reported sleep problems and mean and variability of actigraphy-based sleep measures. SD in sleep efficiency was significantly correlated with caregiver-reported sleep problems (*p* < 0.05).

**TABLE 5:** Results of association analysis between actigraphy measures with caregiver sleep reports, ASD symptoms, and caregiver-reported behaviors using mixed effects model analysis*.

Actigraphy measure	Caregiver Sleep Report (ABI)	ASD Symptoms (SRS-2)	Irritability (ABC)	Hyperactivity (ABC)	Anxiety (ABC)
**Sleep duration**					
Mean	–0.09	–0.08	0.09	0.02	–0.009
SD	–0.05	0.12	–0.08	0.06	0.1
CV	–0.03	–0.02	0.04	–0.004	–0.01
**Sleep start**					
Mean	0.02	0.09	–0.15	–0.13	0.03
SD	–0.02	0.05	–0.06	0.16	0.03
CV	–0.09	0.06	0.003	–0.08	0.07
**Awakenings**					
Mean	–0.03	0.39***	–0.006	−0.28*	0.3**
SD	–0.11	0.25**	–0.02	–0.14	0.1
CV	–0.02	0.78***	0.05	−0.41***	0.43***
**Sleep efficiency**					
Mean	0.11	–0.03	0.04	0.09	0.04
SD	−0.19*	0.14	–0.05	–0.18	–0.02
CV	–0.02	0.56***	0.06	−0.37***	0.29**

*Table shows values of regression coefficients for actigraphy term in the model equation provided in the data analysis section. ABC, Aberrant Behavior Checklist; ABI, Autism Behavior Inventory; ASD, autism spectrum disorder; CV, coefficient of variation; SD, standard deviation; SRS-2, Social Responsiveness Scale 2. *p < 0.05; **p < 0.01; ***p < 0.001 (with FDR adjustment).

### Relationship Between ASD Symptoms and Actigraphy: ASD

Correlations between ASD core symptoms obtained from SRS-2 with mean and variability of actigraphy-based sleep measures are shown in Table 5. Mean and variability of number of awakenings were significantly correlated with SRS-2 scale scores (*p* < 0.001 for mean and CV of number of awakenings; *p* < 0.01 for SD of number of awakenings). CV of sleep efficiency was also significantly correlated with SRS-2 scores (*p* < 0.001).

### Correlation of Caregiver-Reported Internalizing and Externalizing Behaviors With Actigraphy: ASD

The correlations between mean and variability of actigraphy-based sleep measures with caregiver-reported Irritability (ABC), Hyperactivity/Impulsivity (ABC), and anxiety (CASI-Anx) are shown in Table 5. Mean (*p* < 0.05) and CV (*p* < 0.001) of number of awakenings were significantly correlated with Hyperactivity. CV of sleep efficiency was significantly correlated with Hyperactivity (*p* < 0.001). Mean (*p* < 0.01) and CV (*p* < 0.001) of number of awakenings as well as CV (*p* < 0.01) of sleep efficiency were significantly correlated with CASI-Anx.

### Correlation of Caregiver-Reported Sleep With Caregiver-Reported Behaviors (ABI Sleep, CASI-Anx, ABC Hyperactivity, and ABC Irritability): ASD

Significant positive correlations between caregiver-reported sleep problems and all other caregiver-reported behaviors across almost all time points (Table 6) were also observed. Caregiver-reported anxiety showed the strongest correlation with caregiver-reported sleep problems across all time points (baseline *r* = 0.49, midpoint *r* = 0.48, endpoint *r* = 0.53, AMI watch-wearing individuals at midpoint *r* = 0.44, AMI watch-wearing individuals at endpoint *r* = 0.54, all *p* < 0.001).

**TABLE 6:** Spearman correlation coefficients between caregiver-reported sleep problems and caregiver-reported behaviors.

	ADOS CSS Total (baseline only) clinician reported	SRS-2 Total	Hyperactivity (ABC)	Irritability (ABC)	CASI-Anx
Baseline, all individuals (*N* = 139)	−0.07	0.27**	0.39***	0.37***	0.49***
Midpoint, all individuals (*N* = 124)		0.30**	0.34***	*0*.44***	0.48***
Endpoint, all individuals (*N* = 127)		0.37***	0.38***	*0*.43***	0.53***
Midpoint, individuals wearing AMI watch (for ≥ 2 nights) 7 days before (*N* = 58)		0.21	0.32**	0.28*	0.44***
Endpoint, individuals wearing AMI watch (for ≥ 2 nights) 7 days before (*N* = 38)		0.52**	0.42*	0.49**	0.54**

ABI, Autism Behavior Inventory; ABC, Aberrant Behavior Checklist; ADOS CSS, Autism Diagnostic Observation Schedule calibrated severity score; CASI-Anx, The Child Adolescent Symptom Inventory–Anxiety; FDR, false discovery rate; SRS-2, Social Responsiveness Scale 2. *p < 0.05; **p < 0.01; ***p < 0.001 (with FDR adjustment).

### Prediction Model

**FIGURE 3:**
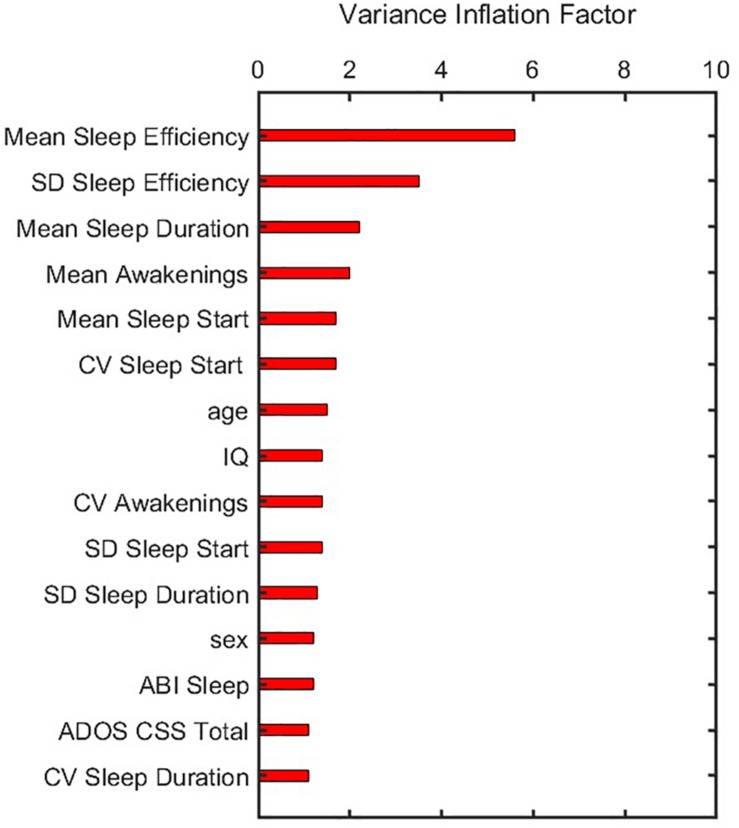
Variance inflation factor of features after they were corrected for multicollinearity and near zero variance. ADOS CSS, Autism Diagnostic Observation Schedule calibrated severity score; CV, coefficient of variation; IQ, intelligence quotient; SD, standard deviation.

**FIGURE 4:**
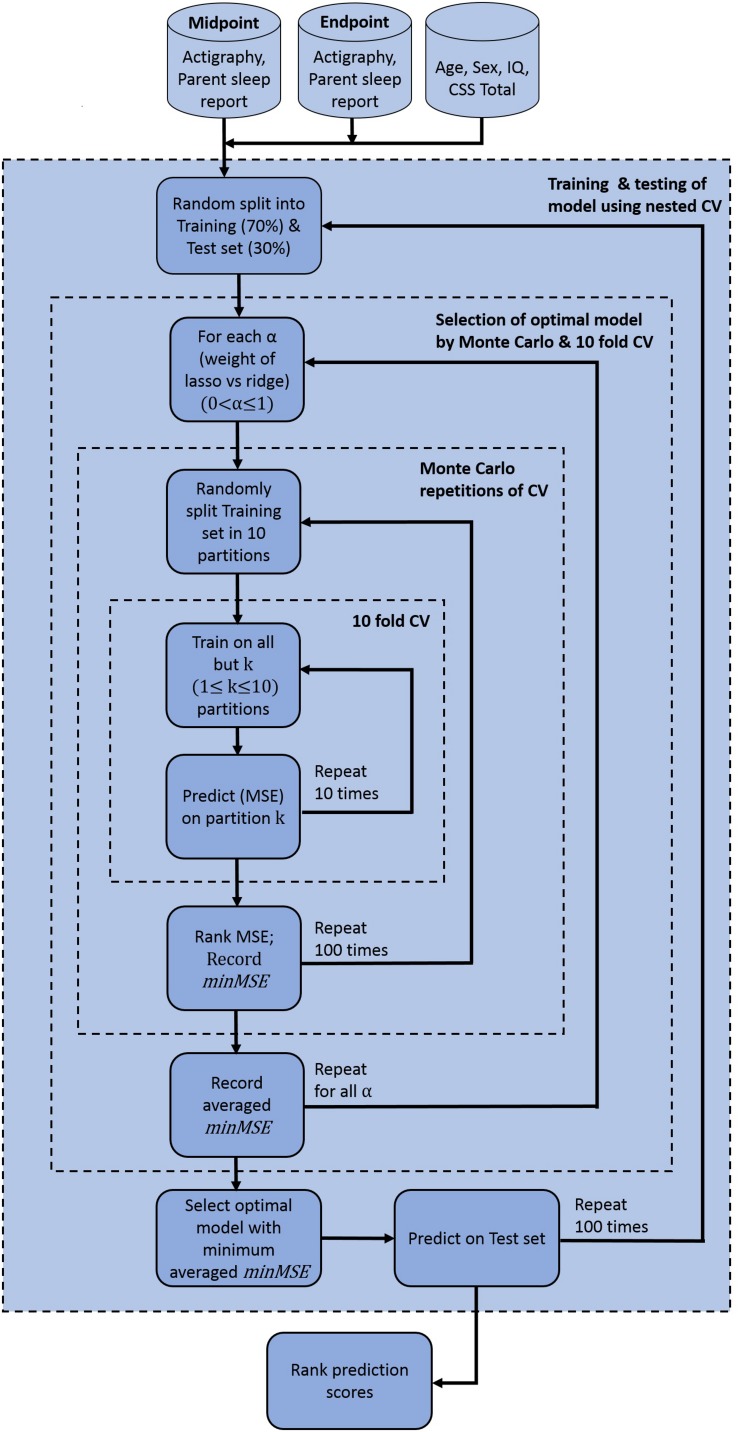
Elastic net model construction and evaluation for anxiety prediction. Model used pre-processed actigraphy measures and ABI sleep items from midpoint and endpoint visits, in addition to age, sex, IQ and ADOS CSS total scores. ABI, Autism Behavior Inventory; ADOS CSS, Autism Diagnostic Observation Schedule calibrated severity score; CV, coefficient of variation; IQ, intelligence quotient; *min*MSE, minimum cross-validated mean squared error; MSE, mean squared error.

In building the prediction model, data were first pre-processed resulting in 15 features that showed variance and were not collinear. Corresponding VIF values after this pre-processing step (Figure 3) demonstrate that all features have values less than a threshold of 10. The elastic net model was thus constructed using these 15 features. A summary of model construction steps and evaluation of performance via elastic net is shown as a schematic in Figure 4, and enumerated below:

**Step 1**: 70% of the participants was randomly split into a training set, and the remaining 30% into a test set.
**Step 2:** In the training set, optimal parameters α and λ were obtained and variable selection was performed through nested cross-validation in the following way:
  (a) For each value of α, 10-fold cross-validation was performed and the minimum cross-validated mean squared error (MSE) was recorded (denoted by *minMSE*, say). The value of α varied from 0 to 1 in increments of 0.05.
  (b) Step a was repeated 100 times. The *minMSE* was then obtained from all 100 repetitions and the average computed.
  (c) The optimal α that corresponded to the minimum averaged *minMSE* was chosen.
**Step 3:** Subset of variables obtained from step 2 was then predicted on the test set, and goodness of prediction of the model was recorded by calculating MSE, R-squared values, and F-statistics. F-statistics tested statistical significance of the model with a constant or intercept-only model.
**Step 4**: Steps 1–3 was repeated 100 times. Thus, prediction was performed on 100 independent test sets and corresponding MSE, R-squared value, and F-statistics were obtained for each case.

**FIGURE 5:**
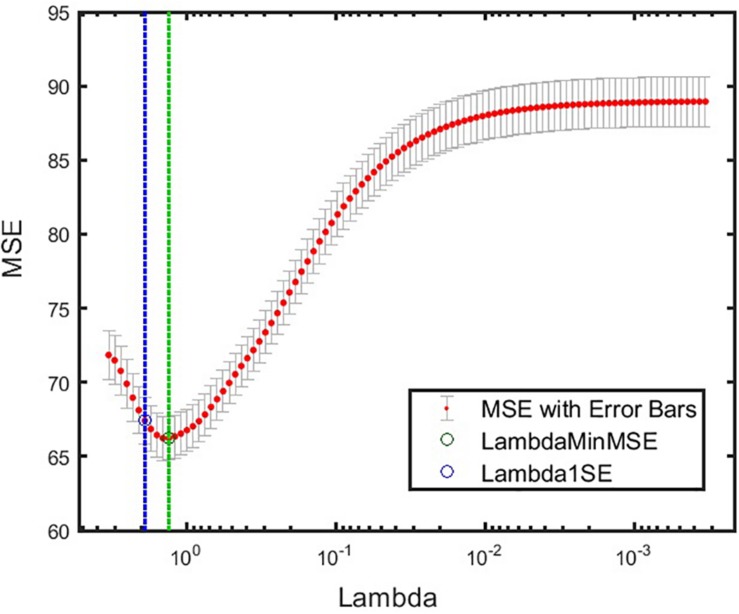
Cross-validated MSE for the best model at various levels of regularization. Green circle and dashed line denote λ with minimum cross-validated MSE. Blue circle and dashed line denote λ with minimum cross-validated MSE plus one standard deviation. α = 1 was obtained for this model. *min*MSE, minimum cross-validated mean squared error; MSE, mean squared error.

The model prediction that explained the highest variance from Step 4 identified the ABI sleep item and variability (SD) of sleep efficiency as the most important predictors of CASI-Anx. The model yielded an R^2^ = 0.51, and a *p* = 8.2 × 10^–9^ F-statistic. The cross-validated MSE of this model is given in Figure 5. Additionally, to interpret the prediction model, a measure of feature importance for each feature was computed to reveal features that drove successful prediction of CASI Total scores. The importance of each feature (*j* = 1,2,…,15) was computed as follows:

$$F\,e\,a\,t\,u\,r\,e\,I\,m\,p\,o\,r\,t\,a\,n\,c\,e_{j}=\frac{\sum_{s=1}^{S}c_{s\,j}*w_{s\,j}}{100}$$

**FIGURE 6:**
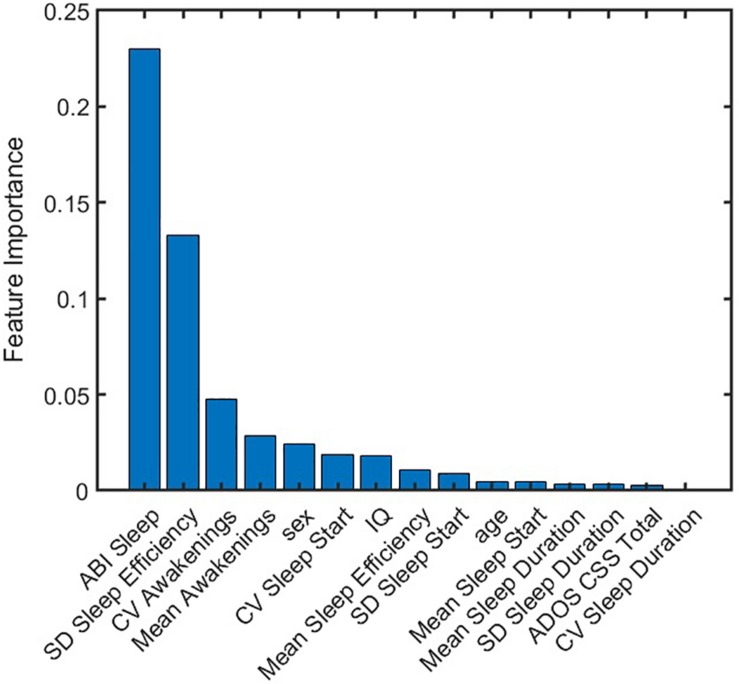
Normalized importance of features on anxiety prediction. Each feature was assigned an importance score from predictions obtained from 100 test sets. ABI sleep item and standard deviation of sleep efficiency were found to be dominant predictors of CASI Total. ABI, Autism Behavior Inventory; ADOS CSS, Autism Diagnostic Observation Schedule calibrated severity score; CASI, Child Adolescent Symptom Inventory; CV, coefficient of variation; IQ, intelligence quotient; SD, standard deviation.

Where, *s* = 1,2,…,100 indicates a test set. *S* represents the total number of test sets, which here is equal to 100. *c*_*sj*_ is an indicator of *j*^*th*^ feature being selected in the *s*^*th*^ test set by the elastic net model. *w*_*s*_ = *r*_s_*f_*s*_ is a function of goodness-of-fit of the model when applied to the *s*^*th*^ test set.*r*_*s*_ denotes the R square value, or variance explained by the model when applied to the *s*^*th*^ test set.*f*_*s*_ is an indicator of significant F-statistic values when applied to the *s*^*th*^ test set.*f*_*s*_ = 1 if *p*-value for F-statistics is less than 0.05, and *f*_*s*_ = 0 if *p*-value for F-statistics is greater than or equal to 0.05. The feature importance score for each feature was then normalized to [0 1] as shown in Figure 6. ABI sleep and SD of sleep efficiency had the highest and second highest feature importance score, respectively. While CV awakenings, mean awakenings, and sex were also important; the importance of the remaining features were almost negligible. Thus, measuring actigraphy sleep measures as well as probing their variability could be important for understanding the role of sleep in anxiety outcomes for individuals with ASD.

## Discussion

The aims of this study were two-fold: First, the relationship between caregiver-reported sleep problems and actigraphy measures of sleep was examined. Second, the relationship between both caregiver-reported and actigraphy measures of sleep and other caregiver-reported behaviors in children and adults with ASD, including symptom severity and internalizing and externalizing behavior, was investigated. In particular, IIV in sleep measures obtained through actigraphy was explored.

A single item on the ABI was used to determine the prevalence of reported sleep problems in the ASD and TD groups. The reported prevalence of 69.1% is consistent with other reports of sleep problems in ASD (Malow et al., 2006; Richdale and Schreck, 2009; Hollway et al., 2013), while reported sleep problems in the TD group was 11.8%. There were no significant differences in caregiver-reported sleep difficulties across the older (≥13 years) and younger age group (<13 years) at baseline (*p* > 0.05), indicating that sleep difficulties are prevalent in both children and adults with ASD.

Using the mixed effects model, caregiver-report of sleep problems was not related to mean actigraphy measures, however, there was a relationship with variability in sleep efficiency. This limited relationship between parent reported sleep and actigraphy in ASD is consistent with previous studies (Goldman et al., 2009; Elrod and Hood, 2015; Veatch et al., 2016), and indicates that actigraphy and caregiver-reported sleep quality, while somewhat related, may explain different aspects of sleep behavior in ASD. Also, the relationship may have been further limited in this study by the use of a single item to report sleep problems. Whilst this might be effective in identifying presence or absence of difficulties, more detail about type of sleep problems (difficulties getting to bed, to sleep, difficulties staying asleep) may have revealed more relationships with the actigraphy measure.

Clinician-reported symptom severity of ASD, using the ADOS, was not related to caregiver-reported sleep problems. However, we did observe relationships between actigraphy and symptom severity on the caregiver-reported SRS, consisting mainly of increased awakenings correlating with increased severity of core ASD symptoms. These awakenings likely reflect poorer quality sleep in some individuals with ASD.

The relationship between caregiver-reported sleep problems and other caregiver-reported behaviors often described in the literature was confirmed, including symptom severity, anxiety, and challenging behaviors (Allik et al., 2006; Malow et al., 2006; Limoges et al., 2013). In addition, our data demonstrated a relationship between both hyperactivity and anxiety, and actigraphy thus removing problems associated with common source or shared method variance. No relationship between irritability and actigraphy sleep measures was found. The demonstration of a relationship between both caregiver-report and actigraphy and behaviors such as hyperactivity and anxiety highlight the potential role of sleep in these behaviors, or vice versa. Interventions which improve sleep in ASD may lead to improvements in symptoms, and similarly, improvements in anxiety could lead to improved sleep quality.

The prediction model showed that actigraphy sleep measures and caregiver-reported sleep problems can help to explain severity of anxiety symptoms in ASD. It further emphasizes the importance of including variability of sleep measures as features when assessing anxiety behaviors (as seen from the high feature importance score of SD of sleep efficiency in Figure 6). As an additional check for robustness of our prediction model, performance on the same dataset, excluding participants who had measurement from both endpoint and midpoint was reassessed. Thus, this dataset only included unique participants, eliminating any potential correlation that may have arisen from multiple visits of the same individual. Training and testing the model, as outlined in Figure 4, also showed that ABI Sleep and SD of sleep efficiency were the predominant predictors of anxiety. This further indicates the robustness of the model, and the importance of not only incorporating actigraphy measures to assess behavioral outcomes but also to compute feature statistics beyond a simple mean value.

### Limitations

All relationships observed in the mixed effect and prediction models are preliminary and need to be confirmed in future studies.

This was an 8 to 10 weeks study and the number of participants who continued to wear the actigraphy watch consistently across the duration of the study was small, and this indicates the practical difficulties in obtaining actigraphy in large scale cohorts, perhaps explaining why there are so few reported studies of this type in ASD. Reduction in wearing the actigraphy device was not found to relate to reported sleep problems, and few caregivers, when asked, reported issues related to tolerability of the device. Participants were required to take off the actigraphy device and replace it with a daytime biosensor in this study (data not reported here). A study we are currently performing uses an alternative actigraphy device, capable of remaining on during the day time and only needing to be removed for charging. The impact of this on ability to obtain consistent actigraphy reports across a 12 weeks interventional study will be assessed.

In addition, a more complete data set would allow investigation of the impact of weekday and weekend observations on sleep measures, which was not possible in the current study, but could influence mean and variability of actigraphy measures.

Given that the current study did not include an intervention, direction of the relationship between sleep and anxiety cannot be assumed. There is support for the role of a bidirectional model in explaining the relationship between sleep and behavior in ASD (Hollway et al., 2013; Adams et al., 2014a). Longitudinal and intervention studies can help to further understand the relationship between sleep and behavior in ASD. For example, short term studies cannot account for the impact of cumulative loss of sleep (Van Dongen et al., 2003). There is evidence that sleep difficulties in childhood are related to psychiatric disorders such as anxiety and depression at a later stage in TD (Shanahan et al., 2014; Steinsbekk and Wichstrøm, 2015) and ASD populations (May et al., 2015; Fletcher et al., 2017). Therefore, it is imperative to assess the relationship between sleep and behavior over time.

Sleep characteristics provided by AMI Motionlogger have been compared to PSG, producing 0.89–0.97% sensitivity, 0.54–0.77% specificity, and 0.87–0.90% accuracy ranges (Rupp and Balkin, 2011; Meltzer et al., 2012). This suggests that actigraphy alone may not provide sufficient measurement of sleep and future combination with other biosignals (e.g., heart rate or heart rate variability) may be required to increase the accuracy of sleep measurement that take place outside of a laboratory. Additionally, it was not possible to obtain information about sleep onset–time taken to fall asleep, from the data obtained in our study, and this has previously been shown to have the highest relationship to parent report of sleep problems in ASD (Lambert et al., 2016).

### Clinical Relevance

The single-item sleep report could prove useful as a screen for sleep difficulties in ASD, and, given the high rate of reported sleep problems in both adults and children, a screening during assessments would be helpful for clinicians to identify triggers for behaviors, with follow up questions as necessary.

The relatively novel measure of IIV was introduced in this study. This variable appears to be useful in explaining some of the differences in caregiver-reported sleep problems and the relationship to caregiver-reported behaviors. In particular, variability in sleep start time correlated with anxiety ratings. Sleep interventions have been shown to reduce IIV in TD individuals, resulting in improvements in daytime functioning and behavior (Bei et al., 2017). Given the relationship observed between sleep variability and anxiety in this study, programs that improve consistency of sleep start time may have a similar impact in ASD. Further, measurement of IIV may prove a useful indicator of program effectiveness alongside other sleep measures. Since the direction of the relationship is not known, changes in sleep measures may be useful as outcomes for observation in programs designed to target anxiety in ASD.

## Summary and Conclusion

A large majority of children and adults with ASD are reported by caregivers to have problems with sleep. These reported problems are strongly related to other caregiver-reported symptoms and behavior in ASD. There is some relationship between actigraphy and caregiver-reported measures, and both contribute to our understanding of the connection between sleep and other behaviors, such as anxiety in ASD. Measuring variability in sleep may prove useful in understanding the relationship between sleep problems and behavior in individuals with ASD. These findings may have implications for both intervention and monitoring outcomes in ASD.

## Data Availability Statement

Publicly available datasets were analyzed in this study. This data can be found here: The data sharing policy of the study sponsor, Janssen Pharmaceutical Companies of Johnson & Johnson, is available at https://www.janssen.com/clinical-trials/transparency. As noted on this site, requests for access to the study data can be submitted through Yale Open Data Access (YODA) Project site at http://yoda.yale.edu.

## Ethics Statement

The studies involving human participants were reviewed and approved by Independent Review Boards (Duke University Health Systems Institutional Review Board, Durham NC and Western Institutional Review Board, Puyallup, WA). The patients/participants provided their written informed consent to participate in this study.

## Author Contributions

AB, MC, NM, SN, DL, AS, MB, MG, GD, RH, BL, FS, AE, and GP, were involved in the study design and/or data collection. MC, DL, AS, and NM were responsible for the statistical analyses. AB, MC, NM, SN, AS, and GP were involved in data analysis. All authors were involved in interpretation of the results and they had full access to all the data in the study and take responsibility for integrity of the data and the accuracy of the data analysis. All authors meet ICMJE criteria and all those who fulfilled those criteria are listed as authors.

## Conflict of Interest

AB, MC, SN, AS, NM, and GP are employees of Janssen Research & Development, LLC and may hold equity. DL was employed by the company Statistically Speaking Consulting. RH received reimbursement for consultation from Janssen Research & Development. GD is on the Scientific Advisory Boards of Janssen Research & Development and Akili, Inc., a consultant to Roche, has received grant funding from Janssen Research & Development and PerkinElmer, and receives royalties from Guildford Press and Oxford University Press. AE received reimbursement for consultation with ProPhase, LLC on their contract with Janssen Research & Development. FS received reimbursement for consultation from Janssen Research & Development and Roche Pharmaceuticals. This study was funded by Janssen Research & Development, LLC. The remaining authors declare that the research was conducted in the absence of any commercial or financial relationships that could be construed as a potential conflict of interest.
